# Modulation of intracellular calcium signaling by microRNA-34a-5p

**DOI:** 10.1038/s41419-018-1050-7

**Published:** 2018-09-27

**Authors:** Caroline Diener, Martin Hart, Dalia Alansary, Vanessa Poth, Barbara Walch-Rückheim, Jennifer Menegatti, Friedrich Grässer, Tobias Fehlmann, Stefanie Rheinheimer, Barbara A. Niemeyer, Hans-Peter Lenhof, Andreas Keller, Eckart Meese

**Affiliations:** 1 Institute of Human Genetics, Saarland University, 66421 Homburg, Germany; 2 Molecular Biophysics, Center for Integrative Physiology and Molecular Medicine, School of Medicine, Saarland University, 66421 Homburg, Germany; 3 Institute of Virology and Center of Human and Molecular Biology, Saarland University, 66421 Homburg, Germany; 4 Institute of Virology and Center of Human and Molecular Biology, Medical School, Saarland University, 66421 Homburg, Germany; 5 Chair for Clinical Bioinformatics, Saarland University, 66123 Saarbrücken, Germany; 6 Center for Bioinformatics, Saarland Informatics Campus, Saarland University, 66123 Saarbrücken, Germany

## Abstract

Adjusting intracellular calcium signaling is an important feature in the regulation of immune cell function and survival. Here we show that miR-34a-5p, a small non-coding RNA that is deregulated in many common diseases, is a regulator of store-operated Ca^2+^ entry (SOCE) and calcineurin signaling. Upon miR-34a-5p overexpression, we observed both a decreased depletion of ER calcium content and a decreased Ca^2+^ influx through Ca^2+^ release-activated Ca^2+^ channels. Based on an in silico target prediction we identified multiple miR-34a-5p target genes within both pathways that are implicated in the balance between T-cell activation and apoptosis including *ITPR2*, *CAMLG*, *STIM1*, *ORAI3*, *RCAN1*, *PPP3R1*, and *NFATC4*. Functional analysis revealed a decrease in Ca^2+^ activated calcineurin pathway activity measured by a reduced IL-2 secretion due to miR-34a-5p overexpression. Impacting SOCE and/or downstream calcineurin/NFAT signaling by miR-34a-5p offers a possible future approach to manipulate immune cells for clinical interventions.

## Introduction

Store-operated Ca^2+^ entry (SOCE) is the central calcium signaling pathway in T cells, regulating cellular activation, proliferation, and migration^1^. Furthermore, apoptosis regulation by intracellular calcium signaling has an important role for negative selection of auto-reactive T cells and T-cell proliferation control during adaptive immune response^2–4^. Upon T-cell receptor (TCR) activation, the second messenger IP_3_ (inositol 1,4,5-trisphosphate) binds to its receptor in the membrane of endoplasmic reticulum (ER) resulting in a depletion of ER Ca^2+^ stores^5–7^. This in turn causes conformational changes and oligomerization of the ER Ca^2+^ sensor STIM (stromal interaction molecule), accompanied by translocation to the plasma membrane^8–10^. Interaction of STIM with Ca^2+^ pore-forming ORAI proteins subsequently induces a concentration-dependent influx of Ca^2+^ ions from extracellular space^11,12^. Within the cell, Ca^2+^ is transported to refill intracellular stores and serves as second messenger^13,14^. Various Ca^2+^-induced pathways, including the calcineurin/nuclear factor of activated T-cell (NFAT) pathway, finally result in transcriptional activation of genes that are essential for T-cell activity^14,15^. Based on its central role for T-cell functions, a deregulation in SOCE is associated with immune deficiency and a reduced anti-tumor immunity in cancer^16–18^.

A recent study on mouse CD4 + T cells possessing an aberrant microRNA biogenesis reveals changes in efficiency of SOCE, suggesting a functional role of miRNAs in SOCE regulation^19^. MicroRNAs (miRs) are small, non-coding RNAs that specifically bind to complementary sequences within the 3′-untranslated region (UTR) of their respective target mRNAs^20,21^. Due to a conjunction with miR-induced silencing complex, miR binding leads to an inhibition of translation or target mRNA degradation and results in a reduction of corresponding protein level^22^. Deregulated microRNAs have a central role for severe diseases including cancer and immune disorders^23,24^. MicroRNA-34a (hsa-miR-34a-5p, miR-34a-5p) appears to have a crucial role in cell function and has been suggested as therapeutic agent in cancer treatment^25–27^. However, a first clinical trial for tumor therapy by miR-34a-5p had to be aborted by reason of detrimental side effects on immune cells^28^. This highlights the general need for a better understanding of the regulatory impact of miR-34a-5p on immune cell function^29^. We recently found expressional changes of miR-34a-5p in T cells of lung cancer patients and miR-34a-5p-mediated targeting of the protein kinase c family, which is central to signaling through TCR and is also described to regulate apoptosis^30–32^. Beside the aforementioned general association of miRs in SOCE regulation and T-cell function, as of now the impact of miRs on SOCE pathway and the downstream calcineurin signaling remains to be further elucidated^33^. Here we demonstrate that miR-34a-5p is a central regulator of store-operated Ca^2+^ signaling and downstream calcineurin/NFAT pathway.

## Results

### MiR-34a-5p overexpression reduces SOCE in Jurkat cells

**Fig. 1:**
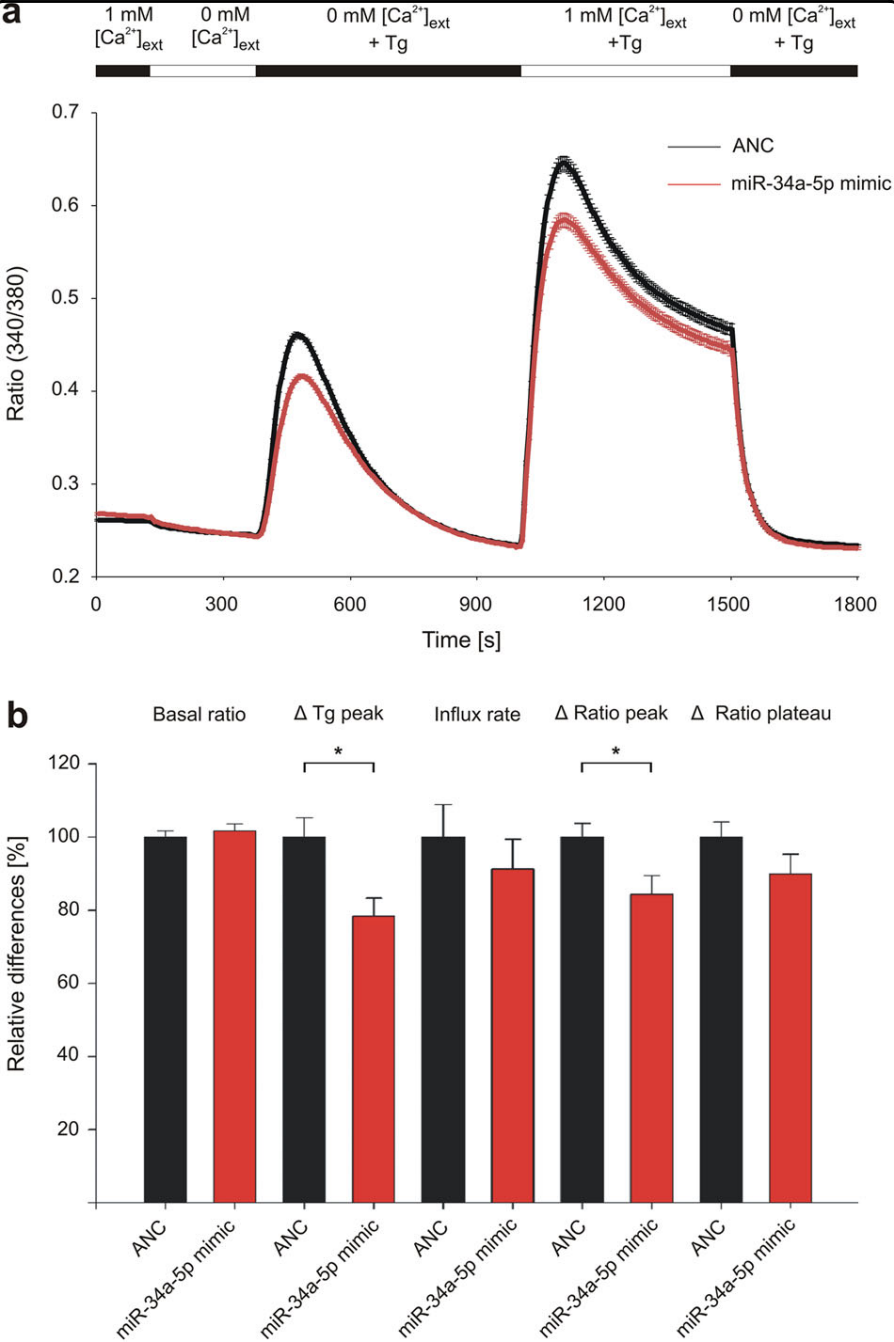
Impact of miR-34a-5p overexpression on store-operated calcium entry in Jurkat cells. Jurkat cells were transfected for 48 h either with non-targeting control (ANC) or synthetic miR-34a-5p mimic. Respective Ca^2+^ imaging was performed in five independent experiments on 3 days of measurement. Intracellular Ca^2+^ was sensed by Fura-2-AM fluorescent dye and SOCE was induced by Thapsigargin (Tg). Cells were perfused with different solutions providing external Ca^2+^ ([Ca^2+^]_ext_). **a** Quotient of Fura-2 fluorescence (Ratio (340/380)) was determined over time of measurement as mean of all tested cells (ANC: *n* = 710; miR-34a-5p: *n* = 756). **b** Ratio (340/380) and delta ratio (340/380) were determined for the functional sections of imaging procedure and evaluated as mean for each of the five experiments. Results were standardized to controls and are shown as means of all experiments with corresponding standard errors (SEM). Statistical evaluation was performed using Student’s *t*-test. A normal distribution of the data was assumed. (*p ≤ 0.05)

To test the impact of miR-34a-5p on SOCE, we analyzed the functional effect of its overexpression on human T-cell line Jurkat. Cells were transfected either with miR-34a-5p mimic or control mimic (AllStars Negative Control, ANC) RNA for 48 h before loading with Fura-2-AM. ER depletion was induced by sarco/endoplasmic reticulum Ca^2+^-ATPase  (SERCA) pump inhibitor Thapsigargin (Tg) and Ca^2+^ release-activated Ca^2+^ (CRAC) influx was initiated, providing external Ca^2+^ solution. As excitation maximum of Fura-2 fluorescent dye changes from *λ* = 380 nm to *λ* = 340 nm, when bound to cytosolic Ca^2+^, relative changes in intracellular calcium concentration were determined by Ratio (340/380) (Fig. 1a).

Statistical evaluation of the functional imaging sections (Fig. 1b) showed no significant effects of miR-34a-5p overexpression on ratio (340/380) in resting cells (basal ratio), as well as in CRAC influx rate and in the plateau phase of CRAC channel activity (Δ ratio plateau). Overexpression of miR-34a-5p however led to a significantly reduced ratio (340/380) for ER depletion by Tg, which was 78.31% compared with control transfected cells. Similar situation was detected for influx peak through CRAC channels (Δ ratio peak), which was reduced to 84.35% compared with control transfected cells.

### MiR-34a-5p targets SOCE and calcineurin/NFAT signaling-related genes

In order to identify target genes of miR-34a-5p related to SOCE and downstream calcineurin signaling, we performed an in silico target prediction. Regulation by miR-34a-5p was analyzed by dual luciferase reporter gene assays. Respective 3′-UTR sequences containing the predicted miR-binding sites were cloned into pMIR-RNL-TK reporter plasmid. Activity of firefly luciferase was measured after 48 h co-transfection of HEK293T cells with microRNA-34a expression plasmids (pSG5-miR-34a) or control (pSG5). Binding of the miR to the 3′-UTR was detected by a decline in relative luciferase activity.

**Fig. 2:**
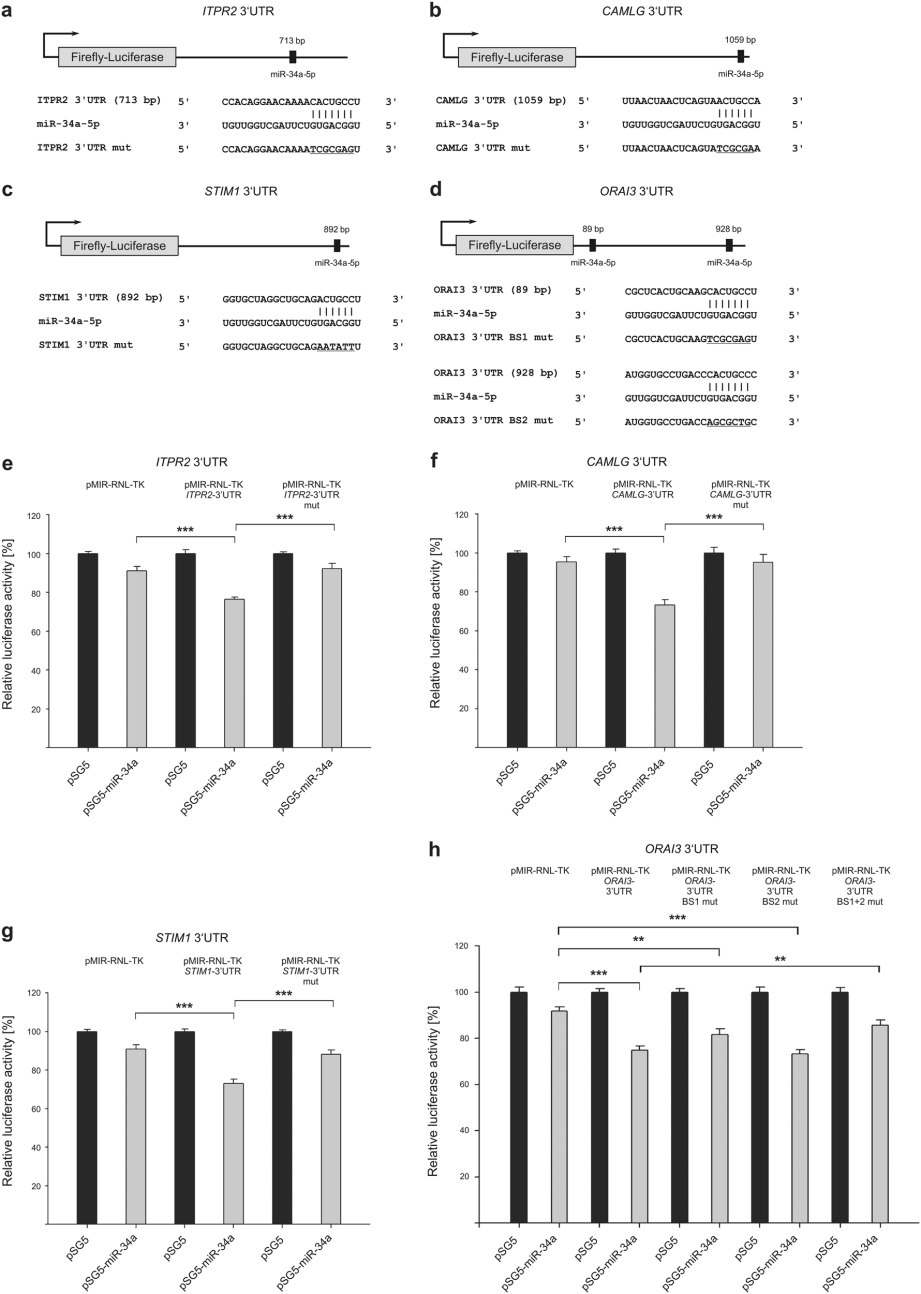
Schematic representation of reporter gene constructs and results of luciferase assays, showing the impact of miR-34a-5p on target genes related to store-operated Ca^2+^ entry. **a**–**d** 3′-UTR sequences of *ITPR2* (inositol 1,4,5-trisphosphate receptor type 2), *CAMLG* (calcium-modulating ligand), *STIM1* (stromal interaction molecule 1), and *ORAI3* (ORAI calcium release-activated calcium modulator 3) were cloned into pMIR-RNL-TK reporter plasmids. The positions of the predicted miR-34a-5p-binding sites within the cloned 3′-UTR reporter constructs are illustrated and denoted. Mutated binding sites are shown underlined. **e**–**h** Relative luciferase activity [%] is shown for empty reporter plasmids (pMIR-RNL-TK), as well as wild type and mutated (mut) 3′-UTR-containing constructs. HEK293T cells were co-transfected with control (pSG5) or miR-34a expression plasmids. Luciferase activities were measured 48 h after transfection. Results are shown as means of four independent experiments with corresponding standard errors (SEM). Statistical evaluation was performed using Student’s *t*-test. A normal distribution of the data was assumed. (*p ≤ 0.05, **p < 0.01, ***p < 0.001; statistical comparison between wild-type 3′-UTR and mutated 3′-UTR containing two miR-34a-5p-binding sites was only performed for double mutated constructs)

Out of 12 predicted and tested miR-34a-5p target genes, 7 were identified being determinably effected by miR-34a-5p. We observed no effect of miR-34a expression on *ITPR1* and *ITPR3* (inositol 1,4,5-trisphosphate receptor 1 and 3), *CALM3* (Calmodulin 3), as well as *ATP2A2* and *ATP2A3* (ATPase sarcoplasmic/endoplasmic reticulum Ca2 + transporting 2 and 3) 3′-UTRs (Supplementary Figures 1 and 2). *ITPR2* (inositol 1,4,5-trisphosphate receptor 2), *CAMLG* (calcium-modulating ligand), *STIM1*, and *ORAI3* (ORAI calcium release-activated calcium modulator 3) were identified as direct target genes of miR-34a-5p with relation to SOCE (Fig. 2). Analysis of *ITPR2*-3′-UTR-containing construct in luciferase assays showed a decrease of relative luciferase activity to 76.32% in cells overexpressing miR-34a. Compared with empty reporter vector, this effect was statistically significant. The miR-34a-5p-specific effect was further confirmed by mutating the predicted binding sequence. The *CAMLG*-3′-UTR construct showed a significantly reduced luciferase activity (73.37%) upon miR-34a overexpression. Again, this effect was statistically significant as compared with empty reporter vector and further confirmed by mutating the predicted binding sequence. *STIM1*-3′-UTR constructs also showed a significant reduced luciferase activity (73.1%), which was reconstituted by mutation of specific miR-34a-5p-binding site. *ORAI3*-3′-UTR construct also led to a significant decline in relative luciferase activity (74.93%) upon miR-34a overexpression. The mutation of single binding sites respectively showed a significant reduction of reporter construct activity by miR-34a overexpression. The relative luciferase activity was reduced to 81.61% for the first binding site and to 73.29% for the second. By testing double mutated constructs, the luciferase activity was reconstituted, showing a combined function of both miR-34a-5p-binding sites.

**Fig. 3:**
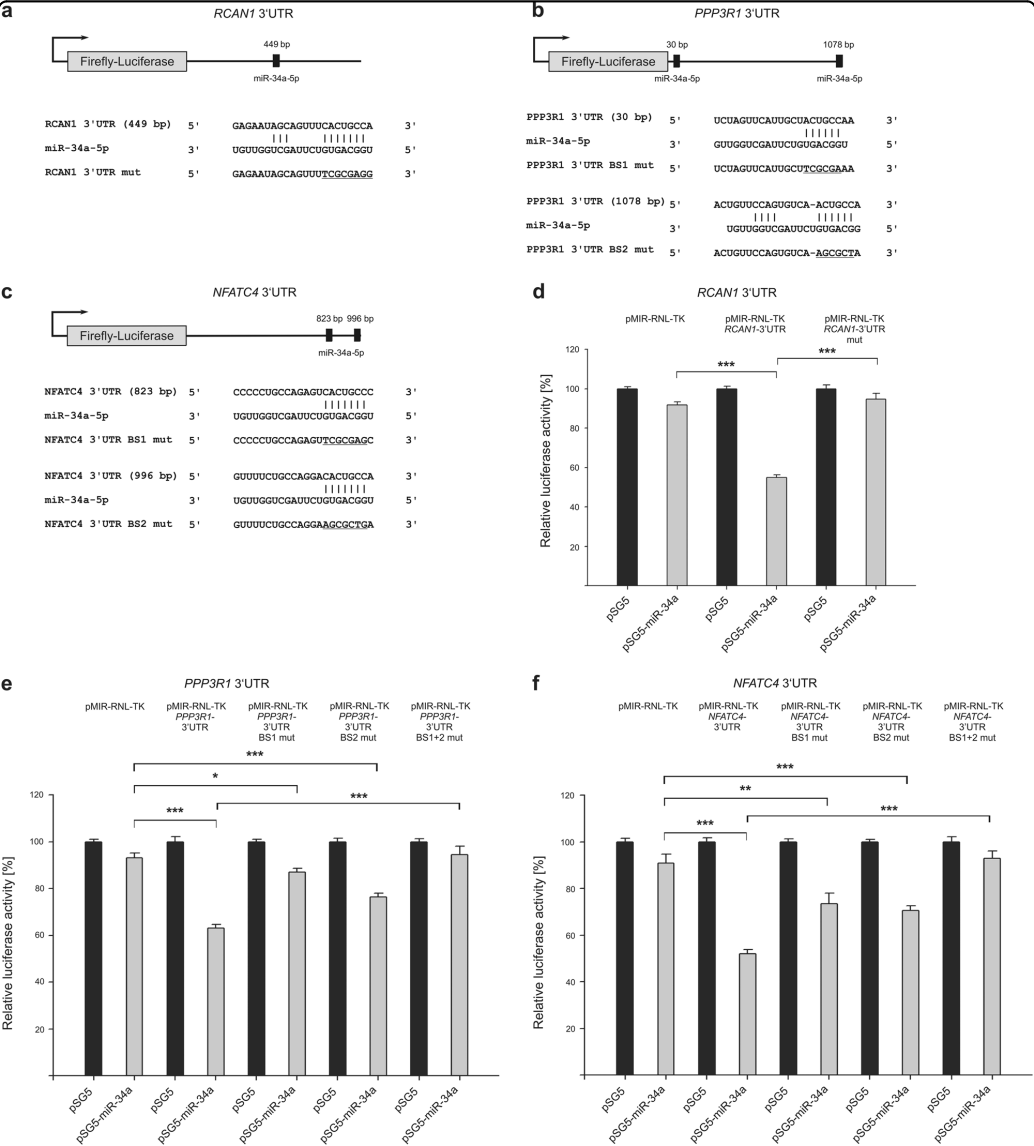
Schematic representation of reporter gene constructs and results of luciferase assays, showing the impact of miR-34a-5p on target genes related to calcineurin/NFAT signaling. **a**–**c** 3′-UTR sequences of *RCAN1* (regulator of calcineurin 1), *NFATC4* (nuclear factor of activated T cells 4), and *PPP3R1* (protein phosphatase 3 regulatory subunit B, alpha) were cloned into pMIR-RNL-TK reporter plasmids. The approximate position of the predicted miR-34a-5p-binding sites within the 3′-UTR reporter constructs are illustrated and the sequences of the binding sites within the respective 3′-UTRs are denoted. Mutated binding sites are shown underlined. **d**–**f** Relative luciferase activity [%] is shown for empty reporter plasmids (pMIR-RNL-TK), as well as wild type and mutated (mut) 3′-UTR-containing constructs. HEK293T cells were co-transfected with control (pSG5) or miR-34a expression plasmids. Luciferase activities were measured 48 h after transfection. Results are shown as means of four independent experiments with corresponding standard errors  (SEM). Statistical evaluation was performed using Student’s *t*-test. A normal distribution of the data was assumed. (*p ≤ 0.05, **p < 0.01, ***p < 0.001; statistical comparison between wild-type 3′-UTR and mutated 3′-UTR containing two miR-34a-5p-binding sites was only performed for double mutated constructs)

*RCAN1* (regulator of calcineurin 1), *PPP3R1* (protein phosphatase 3 regulatory subunit B, alpha), as well as *NFATC4* were further identified as miR-34a-5p target genes important for calcineurin/NFAT signaling (Fig. 3). In comparison with empty reporter plasmid, testing of *RCAN1*-3′-UTR reporter gene construct revealed a reduced activity of luciferase (54.94%). The effect of miR-34a-5p binding again was verified by an increase in reporter gene activity of the mutated construct. Relative luciferase activity of wild-type *PPP3R1*-3′-UTR construct was reduced by miR-34a overexpression to a content of 63.07%. Mutation of binding site one or two respectively within the *PPP3R1* reporter gene construct also led to a significantly reduced luciferase activity to 87.13 and 76.43%, compared with empty control plasmid. Testing of the double mutated construct resulted in a reconstituted activity with significant differences to wild-type 3′-UTR plasmid and verified a functional impact of both binding sites. Analysis of *NFATC4* resulted in similar findings. The effects of miR-34a overexpression on *NFATC4*-3′-UTR constructs led to a reduced relative luciferase activity to 51.96% for wild type, and 73.52% and 70.52% for the respective single mutated constructs. Mutation of both miR-34a-5p-binding sites reconstituted the luciferase activity.

### MiR-34a-5p overexpression reduces endogenous STIM1, PPP3R1, and NFATC4 protein levels

**Fig. 4:**
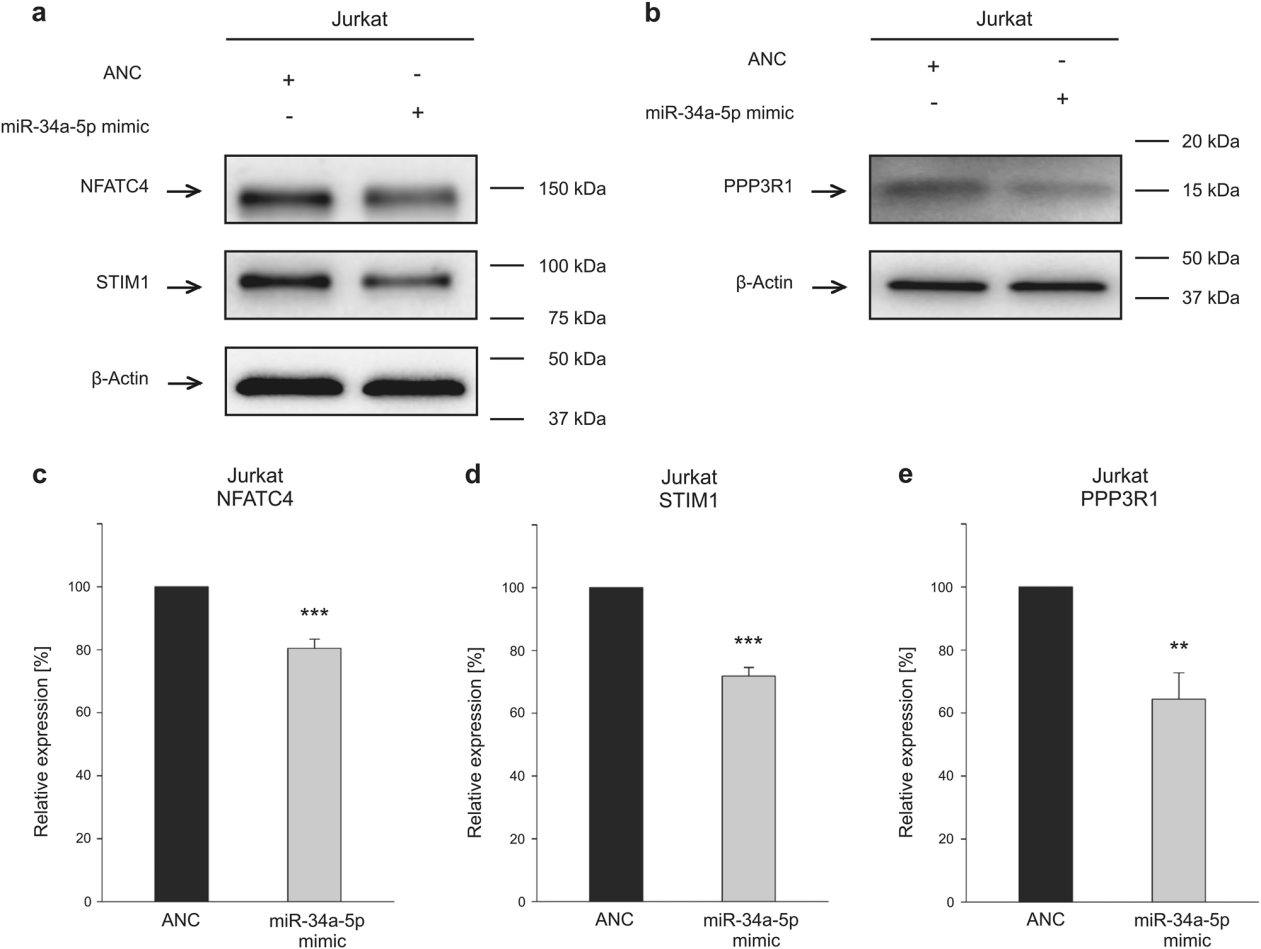
Western blot analysis of endogenous NFATC4, STIM1, and PPP3R1 protein levels in miR-34a-5p-overexpressing Jurkat cells. **a**, **b** Representative western blot images. Jurkat cells were transfected for 48 h either with non-targeting control (ANC) or synthetic miR-34a-5p mimic. NFATC4, STIM1, and PPP3R1 protein was detected by specific monoclonal antibodies in western blot analysis. β-Actin served as loading control. **c**–**e** Quantification of NFATC4, STIM1, and PPP3R1 expression by densitometric analysis of three independent western blot experiments. Respective protein expression was standardized according to β-Actin loading control and expression level of the control transfected cells was set to 100%. Results are shown as means of five independent experiments with corresponding standard errors (SEM). Statistical evaluation was performed using Student’s *t*-test. A normal distribution of the data was assumed. (*p ≤ 0.05, **p < 0.01, ***p < 0.001; PPP3R1 protein phosphatase 3 regulatory subunit B alpha, NFATC4 nuclear factor of activated T cells 4, STIM1 stromal interaction molecule 1

Due to the central roles of STIM, calcineurin, and NFAT in SOCE and calcineurin pathway, respectively, we analyzed endogenous protein level of STIM1, PPP3R1, and NFATC4 upon microRNA-34a overexpression. The abundance of endogenous *NFATC4* and *STIM1* mRNA, as well as transfection efficiency of miR-34a-5p in Jurkat cells were shown by quantitative reverse-transcriptase PCR (RT-PCR) (Supplementary Figure 3). Endogenous protein expression of NFATC4, STIM1, and PPP3R1 was analyzed by western blotting in Jurkat cells (Supplementary Figure 4). Cells were transfected using miR-34a-5p mimic or control mimic (ANC) RNA for 48 h. Proteins were separated by SDS-polyacrylamide gel electrophoresis (PAGE) and the target proteins were identified by specific monoclonal antibodies. Relative protein expression was related to a β-Actin loading control. In Jurkat lymphocytes overexpressing miR-34a-5p, NFATC4 protein level was significantly reduced to 80.39% as a result of five independent experiments. Relative endogenous STIM1 expression was reduced upon miR-34a-5p overexpression to 71.79% and PPP3R1 protein level was reduced to 64.40% (Fig. 4).

### MiR-34a-5p overexpression reduces IL-2 production in Jurkat cells

**Fig. 5:**
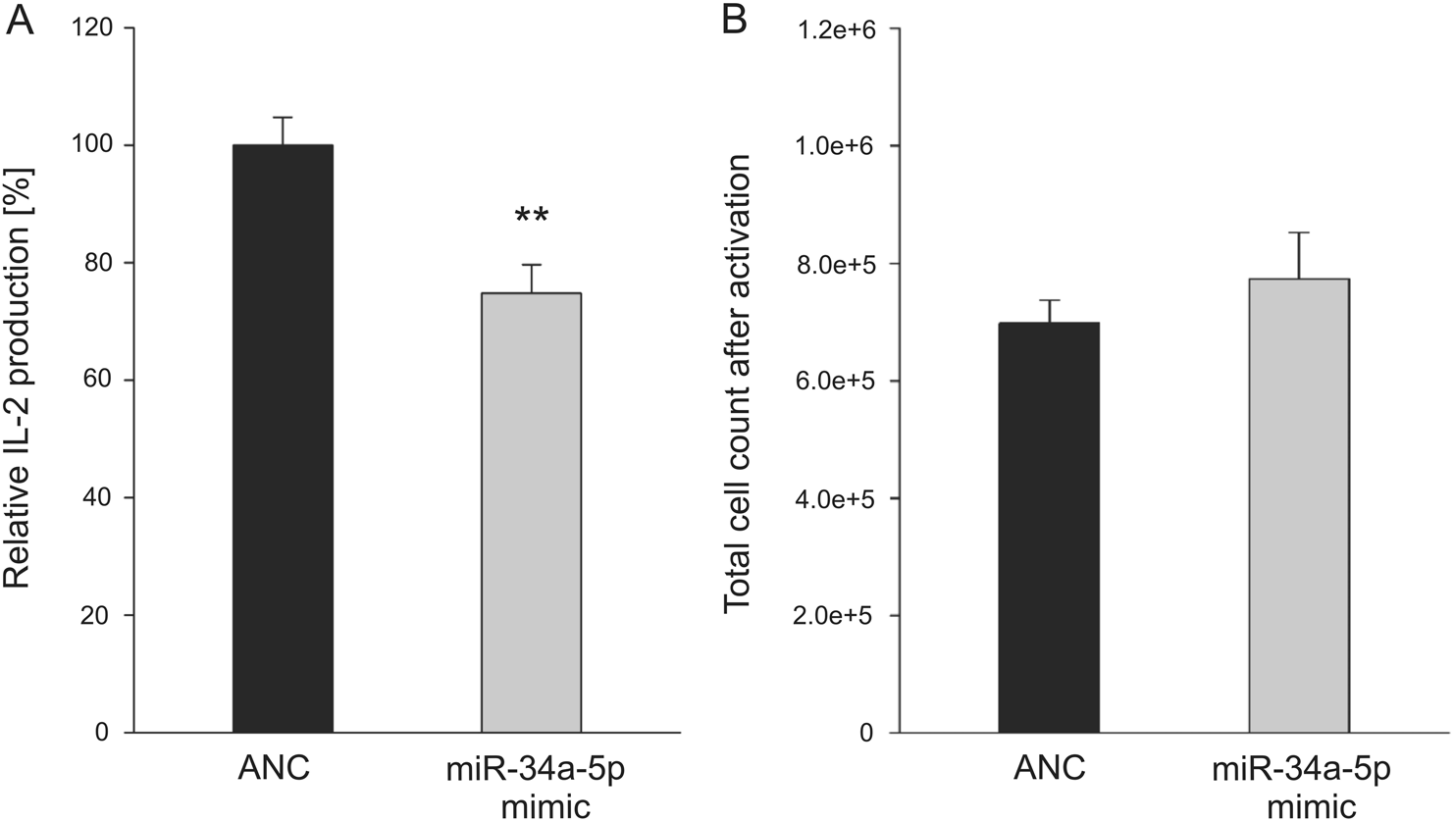
IL-2 expression of miR-34a-5p-overexpressing cells. Jurkat E6.1 cells were transfected for 48 h either with non-targeting control (ANC) or synthetic miR-34a-5p mimic. Twenty-four hours post transfection, a count of 3.5 × 10^5^ cells was activated for IL-2 production by CD2/CD3/CD28 beads and PMA. **a** IL-2 production was quantified by ELISA of the respective supernatants for a total of five independent experiments. Results of IL-2 production were standardized according the control transfected cells. **b** Corresponding cell counts were determined, when supernatants were collected. Results are shown as means with corresponding standard errors (SEM). Statistical evaluation was performed using Student’s *t*-test. A normal distribution of the data was assumed. (*p ≤ 0.05)

Toward a functional analysis of miR-34a-5p overexpression in calcium induced T-cell activation, we analyzed calcium–calcineurin signaling regulated production of cytokine interleukin-2 (IL-2) in Jurkat cell line E6.1. To this end, Jurkat E6.1 cells were activated by αCD2/αCD3/αCD28 beads and phorbol 12-myristate 13-acetate (PMA) subsequent to transfection with ANC or miR-34a-5p mimic. The total number of the activated, IL-2-producing cells was determined and IL-2 quantification in the supernatants was performed by enzyme-linked immunosorbent assay (ELISA) 24 h after activation in five independent experiments (Fig. 5). MiR-34a-5p overexpression reduced IL-2 production to 74.78% as compared with control transfected cells. The mean count of miR-34a-5p-transfected cells was slightly but not significantly increased (7.74 × 10^5^ cells) as compared with control transfected cells (6.98 × 10^5^ cells). The same was true for the counts of non-activated Jurkat cells 48, 72, or 96 h post transfection (Supplementary Figure 5).

### MiR-34a-5p overexpression reduces calcium influx in T-cell activation signaling of Jurkat cells

**Fig. 6:**
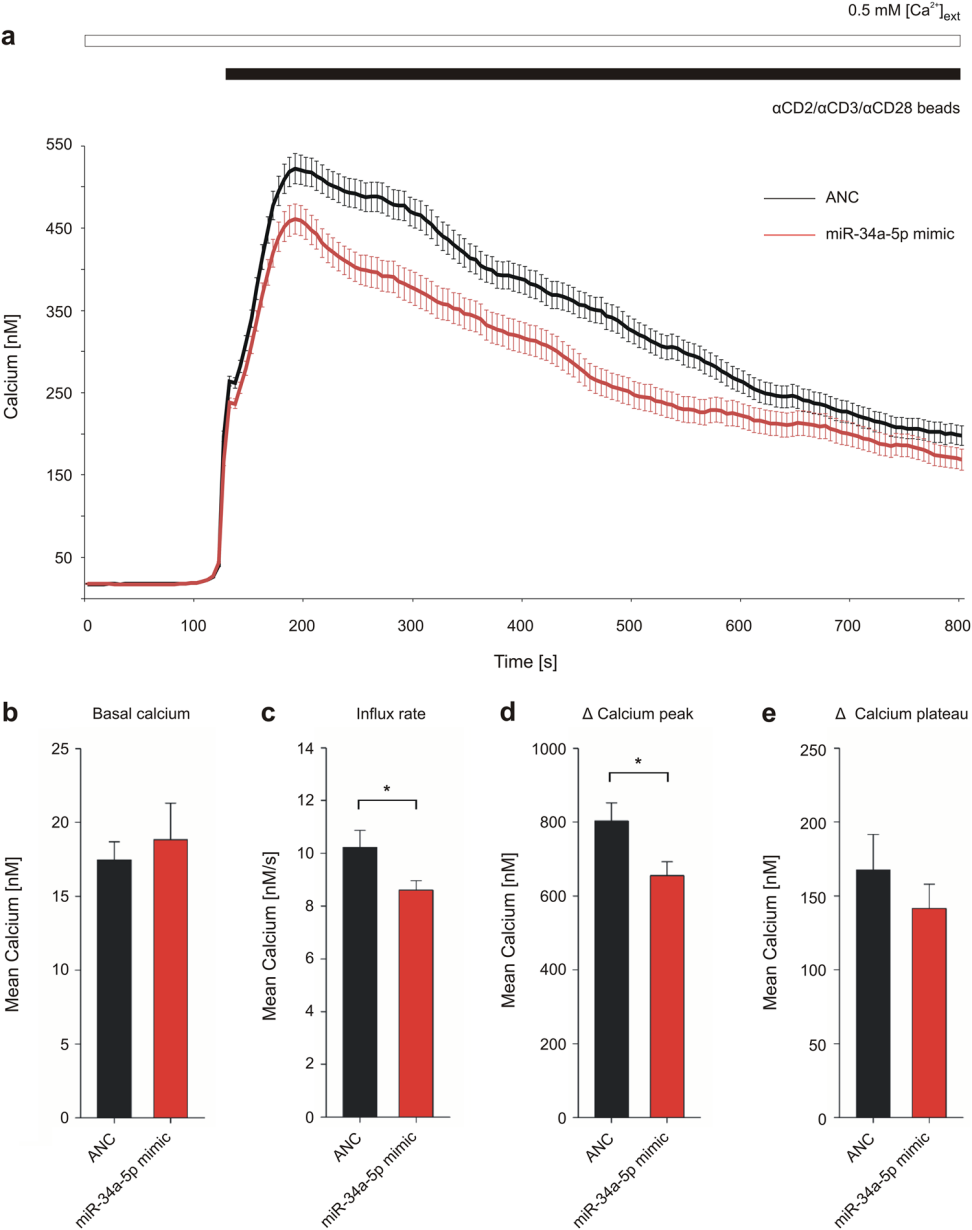
Impact of miR-34a-5p overexpression on calcium signaling in Jurkat cell activation. Jurkat cells were transfected for 48 h either with non-targeting control (ANC) or synthetic miR-34a-5p mimic. Respective Ca^2+^ imaging was performed in eight independent experiments on 3 days of measurement. Intracellular Ca^2+^ was sensed by Fura-2-AM fluorescent dye and SOCE was induced by αCD2/αCD3/αCD28 beads, while providing external Ca^2+^ solution. **a** Intracellular calcium concentration was determined over time of measurement and is shown as mean of all tested cells (ANC: *n* = 407; miR-34a-5p: *n* = 277). **b**–**e** Functional sections of the imaging procedure were evaluated as mean for each of the eight experiments respectively and are shown as mean for all experiments with corresponding SEM. Statistical evaluation was performed using Student’s *t*-test. A normal distribution of the data was assumed. (*p ≤ 0.05)

To further confirm the effect of miR-34a-5p overexpression on calcium signaling, calcium imaging was performed for Jurkat cells upon stimulation by αCD2/αCD3/αCD28 beads (Fig. 6). Relative changes in intracellular calcium concentration were determined by ratio (340/380) of Fura-2-AM fluorescent dye and absolute calcium concentrations were determined by calibration of Fura-2 Ratio within wild-type cells. No significant effects of miR-34a-5p overexpression were observed on basal calcium or within the plateau phase of SOCE (Δ calcium plateau). Overexpression of miR-34a-5p, however, led to a significantly reduced Ca^2+^ influx rate induced by bead contact (8.61 nM Ca^2+^/s). In comparison, influx rate of cells transfected by negative control RNA was 10.23 nM Ca^2+^/s. Overexpression of miR-34a-5p led to a reduced intracellular calcium peak after the influx phase as well. In detail, the Δ calcium peak of cells transfected by miR-34a-5p mimic was significantly reduced (655.45 nM) as compared with control cells showing a Δ peak of 803.11 nM.

## Discussion

MiR-34a-5p is involved in regulation of cell cycle, migration, differentiation, and apoptosis^25^. We recently showed that miR-34a targets several members of the protein kinase C family, which functions in Ca^2+^ signaling through TCR. We also found significantly elevated miR-34a-5p expression in CD3 + T cells of lung cancer patients^30,31^. Here we show that miR-34a-5p is a key regulator of SOCE and calcineurin signaling. In the following, we address the miR-34a-5p targets and the effects of a downregulation of these targets.

The most upstream miR-34a-5p target gene we identified of SOCE pathway was *ITPR2*. *ITPR2* is encoding for IP3 receptor (IP3R) type 2, which is located in the ER membrane^34,35^. Among the three types of IP_3_Rs that are expressed in T cells (*ITPR1*, *ITPR2*, and *ITPR3*), type 2 shows the highest sensitivity to IP_3_^34–36^. It is conceivable that the activation of IP3Rs via TCR first leads to a depletion of ER Ca^2+^ stores and the induction of SOCE^5–7^, followed by a period of reduced responsiveness mediated by miR-34a-5p that downregulates *ITPR2*.

The second gene, which we identified as a target gene of miR-34a-5p encoding an ER membrane protein, was *CAMLG*. Overexpression of CAMLG in Jurkat cells causes IP_3_-independent influx of extracellular Ca^2+^ and enables NFAT-controlled transcription events^37,38^. CAMLG-depleted CD8 + positive T cells show a reduced ability of target cell destruction^39^. A miR-34a-5p-dependent reduction of CAMLG protein level may therefore be associated with a reduced cytosolic Ca^2+^ concentration and a decrease in NFAT-mediated transcription to regulate T-cell function.

ER Ca^2+^ depletion leads to activation of CRAC channels^10^. The CRAC consist of the Ca^2+^ sensor STIM, encoded by two different homologs in mammals (*STIM1* and *STIM2*), and the pore-forming ORAI in plasma membrane, with three different mammalian homologs (*ORAI1*, *ORAI2*, and *ORAI3*)^10,40–42^. The ER Ca^2+^ sensor STIM1 is the third ER membrane protein that we identified as a target of miR-34a-5p. In conjunction with ORAI1, STIM1 is the primary component of the CRAC and enables proper SOCE function^18,41^. Deletion of STIM1 in mouse T cells is associated with a reduction in NFAT-regulated transcription and cytokine expression^43^. STIM1 mutations with a loss of STIM1 function result in the absence of SOCE and severe immune deficiency in human^17,18^. In addition, we identified *ORAI3* as a direct target gene of miR-34a-5p. ORAI3 forms homomeric or heteromeric channels in the plasma membrane^44^. Involvement of ORAI3 in channel formation leads to a reduced Ca^2+^ entry, when compared with homomeric ORAI1 channels^44,45^. An increased ORAI3 expression within an inflammatory environment has been associated with a reduced sensitivity of effector T cells to reactive oxygen species^46^. Based on these data, the posttranscriptional reduction of ORAI3 by miR-34a-5p is likely to be associated with an increased ROS sensitivity of effector T cells.

Analyzing the impact of miR-34a-5p overexpression on SOCE functionality by Tg-induced Ca^2+^ signaling revealed both a decreased ER depletion and a decreased maximum Ca^2+^ influx through CRAC channels. The same was true for a more physiological stimulation of calcium signaling by αCD2/αCD3/αCD28 beads. This led to a decreased calcium influx rate composed of the initial ER depletion and subsequent CRAC channel activation. In addition, the overexpression of miR-34a-5p reduced the maximum of Ca^2+^ influx. Besides IP3Rs, which are known to be responsible for ER calcium leak and ER depletion^47^, CAMLG is likely to act on ER depletion as a potential ER leak channel or a SERCA inhibitor^37^. Hence, the reduced ER depletion may be due to the downregulation of *ITPR2* and *CAMLG* in miR-34a-5p-overexpressing cells. The reduced calcium entry of extracellular calcium in SOCE may result from STIM1 reduction in miR-34a-5p-overexpressing cells. Analysis of Jurkat cells with a reduced STIM1 expression by small interfering RNA also showed a decreased calcium influx through store-operated channels^48^. As ORAI3 involvement in Ca^2+^ channel formation leads to a reduced Ca^2+^ entry^44,45^, a reduced ORAI3 expression in miR-34a-5p-overexpressing cells should, however, result in an increase in SOCE. ORAI3 regulation by miR-34a-5p may impact SOCE to a lower degree than the miR-34a-5p-mediated STIM1 reduction, which regulates activity of all ORAI homologs. Nevertheless, the opposite effect of ORAI3 downregulation may partially diminish the effect caused by a reduced STIM1 expression upon miR-34a-5p overexpression.

Cytosolic Ca^2+^ serves as second messenger that induces pathways such as calcineurin/NFAT signaling ^14,15^. Within the calcineurin/NFAT pathway, we and others identified *RCAN1* as a miR-34a-5p target gene^49^. Depending on the RCAN phosphorylation status^50^, a reduced level of RCAN1 protein as a result of miR-34a-5p binding may lead to either an inhibition or an enhancement of calcineurin/NFAT signaling.

The second identified miR-34a-5p target gene of calcineurin/NFAT pathway was *PPP3R1*, which encodes calcineurin subunit B. Heteromeric calcineurin consists of a catalytic calcineurin A subunit and a regulatory B subunit^51^. Calcineurin B binds to intracellular Ca^2+^ and induces structural changes of calcineurin A^52,53^. Activation of calcineurin A subunit by cytosolic calcium sensor calmodulin subsequently facilitates dephosphorylation of transcription factor NFAT, resulting in nuclear NFAT translocation and enhanced transcription of T-cell-activating genes^53–56^. Moreover, calcineurin B prevents degradation of calcineurin A^57^. Posttranscriptional downregulation of *PPP3R1* gene by miR-34a-5p may therefore impact phosphatase activity as well as stability of the calcineurin complex. The third miR-34a-5p target gene within the calcineurin/NFAT pathway, we identified by luciferase assays and western blotting, was *NFATC4* (NFATC4/NFAT3). NFAT proteins constitute a family of transcription factors that enhance transcription of genes, which are crucial for T-cell activity^55,56,58^. Although there were some reports suggesting that NFATC4 is not expressed in T lymphocytes^59–61^, we and others showed NFATC4 expression in T lymphocytes^62,63^. Downregulation of NFATC4 has been shown to be associated with effective cytokine expression in human CD4 + T cells^63^, suggesting that regulation of NFATC4 expression by miR-34a-5p may take part in orchestrating this process.

We found that miR-34a-5p overexpression reduced IL-2 production. IL-2, which is known to be regulated by SOCE and NFAT/calcineurin signaling, is secreted during immune response and stimulates activation, proliferation, and effector functions in an auto- and paracrine manner^64–66^. The reduced secretion of IL-2 upon miR-34a-5p overexpression is likely to be mediated by the downregulation of the miR-34a-5p target genes verified within SOCE and calcineurin pathway. There may also be an impact on IL-2 transcription due to further miR-34a-5p targets and additional signaling cascades^67^.

T-cell apoptosis is regulated by calcium signaling and has an important role for the negative selection of auto-reactive T cells and the control of proliferating T cells during adaptive immune response^2–4^. Besides the aforementioned effects on T-cell activation, calcium regulation by miR-34a-5p may impact cellular viability^68,69^. Depletion of ER Ca^2+^ and its uptake into mitochondria are connected to intrinsic apoptosis pathway^68,70^. Extrinsic apoptosis signaling is initiated by secretion of fas ligand binding to fas receptors in a para- or an autocrine manner^2,71,72^. Expression of fas ligand is promoted by a depletion of ER calcium and is transcriptionally controlled by calcineurin/NFAT pathway^73,74^. Targeting of *ITPR2* and *STIM1*, as well as calcineurin coding *PPP3R1* and *NFATC4* by miR-34a-5p may therefore exert an anti-apoptotic effect through a reduced ER depletion and the downregulation of FAS/FASLG signaling. By contrast, a reduced protein level of CAMLG due to miR-34a-5p expression may lead to pro-apoptotic effects, as CAMLG has been shown to prevent cell death in activated T cells^39^. Hence, the complex regulatory feedback loop of miR-34a expression, immune cell activation, and apoptosis^75,76^ may be influenced by a balance of pro- or anti-apoptotic target genes of calcium signaling cascade, which in turn effects the cellular viability during adaptive immune response.

Taken together, miR-34a-5p overexpression leads to an inhibition of store-operated Ca^2+^ signaling and impacts downstream calcineurin/NFAT signaling by targeting specific related genes. Analyzing Jurkat cells as a model for T-cell activation, we observed reduction of SOCE and IL-2 secretion by miR-34a-5p overexpression. An increased miR-34a-5p expression may therefore negatively impact activation, proliferation, effector functions, as well as survival of T cells. These data and the previously observed overexpression of miR-34a-5p in T lymphocytes of cancer patients^30,31^ are possibly indicating an inhibition of immune cell function by miR-34a-5p in the anti-tumor immune response. Our results underline the need for a deeper understanding of the effects of miR-34a-5p overexpression on T-cell activation signaling. Further analyses will help to reveal the impact of miR-34a-5p overexpression on primary human T cells including different T- cell subtypes.

## Materials and methods

### Cell lines

Human Jurkat and HEK293T cell lines were purchased from the Leibniz Institute DSMZ-German collection of microorganisms and cell cultures. IL-2-producing Jurkat cell line E6.1 was purchased from European Collection of Authenticated Cell Cultures (Salisbury, UK). HEK293T cells were cultured in Dulbecco’s modified Eagle’s medium (Life Technologies GmbH, Darmstadt, Germany) and both Jurkat subtypes were cultured in RPMI 1640 medium (Life Technologies GmbH), respectively, supplemented with 10 % fetal bovine serum (Biochrom GmbH, Berlin, Germany), penicillin (100 U/mL), and streptomycin (100 μg/mL). Cells were passaged for less than 3 months after receipt.

### Tg-induced Ca^2+^ imaging analysis of miR-34a-5p-overexpressing Jurkat cells

Jurkat cells/well (2.5 × 10^5^) were seeded out in six-well plates and were transfected either with ANC or with syn-hsa-miR-34a-5p miScript miRNA Mimics (QIAGEN N.V., MIMAT0000255: 5′-UGGCAGUGUCUUAGCUGGUUGU-3′), complying with HiPerFect™ transfection reagent protocol (Qiagen, Hilden, Germany). Transfected cells were incubated for overall 48 h before Ca^2+^ imaging. Cells were loaded with 1 μM Fura-2-AM (Invitrogen, Waltham, MA, USA) for 25 min at room temperature and fixed on poly-ornithine-coated glass coverslips. For imaging procedure, cells were perfused with different solutions, providing external Ca^2+^. External Ca^2+^ solution (1 mM) contained 155 mM NaCl, 2 mM MgCl_2_, 10 mM glucose, 5 mM Hepes, and 1 mM CaCl_2_. In 0 mM Ca^2+^ solution, CaCl_2_ was replaced by 1 mM EGTA and 3 mM MgCl_2_ (pH 7.4 with NaOH). SOCE was induced using 1 μM irreversible SERCA inhibitor Tg (Invitrogen). Fura-2 fluorescence (F) was detected alternating its excitation from *λ* = 340 nm (Ca^2+^ bound, F_340_) to 380 nm (Ca^2+^ free, F_380_). Images were analysed by TILLVision software (TILL Photonics GmbH, Gräfelfing, Germany). Ratio (340/380) was determined by quotient F_340_/F_380_. Jurkat cells with basal calcium of > 0.4 ratio (340/380) were excluded from analysis as pre-activated. Ratio (340/380) was determined for the functional sections of imaging procedure using IGOR Pro (WaveMetrics) software. For delta ratio, the minimum before adding the Tg was subtracted from Tg peak (ΔTg peak), ratio (340/380) before adding 1 mM Ca^2+^ solution was subtracted from ratio (340/380) maximum and plateau, respectively (Δ ratio peak, Δ ratio plateau). Influx rate was determined by the slope of ratio, when adding the 1 mM Ca^2+^ solution.

### Ca^2+^ imaging analysis of activated Jurkat cells overexpressing miR-34a-5p

Jurkat E6.1 cells were transfected with ANC or miR-34a-5p mimics for a period of 48 h and utilized for calcium imaging as described above. Calcium signaling was stimulated by MACSiBead™ particles from human T Cell Activation/Expansion Kit (Miltenyi Biotec GmbH, Bergisch Gladbach, Germany). External calcium was provided by a 0.5 mM Ca^2+^ solution. Absolute intracellular Ca^2+^ concentration was determined by the in situ calibration of Fura-2-AM and calculated from [Ca^2+^]_intracellular_ = K*(Ratio − Ratio_min_)/(Ratio_max_ − Ratio)^77^. At least 20 cells per measurement were chosen for calcium analysis by the criteria of a bead contact. The functional data were again analyzed with the software IGOR Pro (WaveMetrics). For calculation of delta calcium, basal calcium was subtracted from calcium peak (Δ calcium peak) or from calcium plateau (Δ calcium plateau), respectively.

### Target prediction and assembly of 3′-UTR reporter gene constructs

We used miRWalk 2.0 (http://zmf.umm.uni-heidelberg.de/apps/zmf/mirwalk2/index.html) for an in silico prediction of miR-34a-5p-binding sites within the 3′-UTR of possible target genes^78^. For that purpose, all through miRWalk 2.0 available databases were independently selected to find putative 3′-UTR target sides for seed binding, with a minimum of a 6 nt seed sequence. Results were filtered for a prediction by more than four databases. Subsequently, we chose predicted target genes related to SOCE or calcineurin/NFAT signaling. The respective 3′-UTR sequences of predicted miR-34a-5p target genes were amplified by PCR using specific primer pairs (Supplementary Table 1) and cloned into the multiple cloning site of pMIR-RNL-TK plasmid^79^, using SpeI and SacI restriction sites. Jurkat cDNA was used for template. The denoted parts of human GRCh38/hg38 genome have been cloned utilizing the declared NCBI sequences for reference: *ATP2A2* 3ʹ-UTR (chr12:110,347,119–110,348,110; NM_170665.3), *ATP2A3* 3ʹ-UTR (chr17:3,923,898–3,924,783; NM_005173.3), *CALM3* 3ʹ-UTR (chr19:46,609,301–46,610,572; NM_005184.3), *CAMLG* 3ʹ-UTR (chr5:134,750,951–134,752,087; NM_001745.3), *ITPR1* 3ʹ-UTR (chr3:4,846,620–4,847,798; NM_001168272.1), *ITPR2* 3ʹ-UTR (chr12:26,337,690–26,338,767; NM_002223.3), *ITPR3* 3ʹ-UTR (chr6:33,695,799–33,696,154; NM_002224.3), *NFATC4* 3ʹ-UTR (chr14:24,378,485–24,379,520; NM_001136022.2), *ORAI3* 3ʹ-UTR (chr16:30,953,784–30,954,826; NM_152288.2), *PPP3R1* 3ʹ-UTR (chr2:68,179,165–68,180,278; NM_000945.3), *RCAN1* 3ʹ-UTR (chr21:34,516,824–34,517,736; NM_004414.6), *STIM1* 3ʹ-UTR (chr11:4,092,219–4,093,198; NM_001277961.1). MiR-34a-5p-binding sites were mutated by technique of overlap extension PCR using specific primers^80^ (Supplementary Table 2).

### Dual luciferase reporter gene assays

HEK293T cells were seeded out to a count of 6.5 × 10^4^ cells/well of a 24-well plate. The day after seeding, cells were transfected with 0.8 μg pSG5 or pSG5-miR-34a expression plasmid, respectively, and either 0.2 μg 3′-UTR reporter construct or empty pMIR-RNL-TK plasmid. Transfection was performed following the instructions of Polyfect™ reagent protocol (Qiagen). Forty-eight hours after transfection, cells were lysed and extracts were measured referring to the protocol of Dual Luciferase® Reporter Assay System (Promega, Mannheim, Germany)

### RNA isolation and quantitative real-time PCR

For analysis of gene expression in Jurkat cells, mRNA levels of *ITPR1*, *ITPR2*, *ITPR3*, *ORAI1*, *ORAI2*, *ORAI3*, *STIM1*, *STIM2*, *NFATC4*, and miR-34a-5p were quantified by RT-PCR. Untreated Jurkat cells were lysed using Qiazol (Qiagen) and total RNA was isolated by miRNeasy Mini KIT (Qiagen), following the manufacturer’s protocol. Total RNA (150 ng) was reverse transcribed by miScript RT II Kit into cDNA. cDNA for subsequent miR quantification was transcribed using miScript HiSpec Buffer (Qiagen), for later mRNA quantification miScript HiFlex Buffer (Qiagen) was used. RNU48 and GAPDH served as endogenous control. Expression levels were analysed by miScript PCR System (Qiagen) and a StepOnePlus Real-Time PCR System (Applied Biosystems, Foster City, USA) following the manufacturer’s instructions. Specific primer pairs (QuantiTect Primer Assays) were also purchased from Qiagen: GAPDH (Catalog number QT00079247), *ITPR1* (Catalog number QT00056490), *ITPR2* (Catalog number QT01336468), *ITPR3* (Catalog number QT00011865), miR-34a (Catalog number MS00003318), *NFATC4* (NFATC4_1: QT00013587; NFATC4_2: QT01873326), *ORAI1* (Catalog number QT00202587), *ORAI2* (Catalog number QT00215229), *ORAI3* (Catalog number QT00231910), RNU48 (Catalog number MS00007511), *STIM1* (Catalog number QT00083538), and *STIM2* (Catalog number QT00023744).

### Western blot analysis of endogenous NFATC4, STIM1, and PPP3R1 protein expression in Jurkat and cells

To obtain endogenous protein extracts from Jurkat cells, 2.5 × 10^5^ cells/well were seeded out in six-well plates and were transfected either with ANC or with syn-hsa-miR-34a-5p miScript miRNA Mimics (QIAGEN N.V., MIMAT0000255: 5′-UGGCAGUGUCUUAGCUGGUUGU-3′), complying with HiPerFect™ transfection reagent protocol (Qiagen). Further analysis was performed after an incubation time of 48 h. The transfected cells were lysed by 2 × lysis buffer (130 mM Tris/HCl, 6% SDS, 10% 3-Mercapto-1,2-propanediol, 10% glycerol) and sonification. Whole-cell protein extracts (15 μg) were separated by SDS-PAGE on Mini-Protean^®^ TGX Stain-Free^TM^ Precast Gels (Bio-Rad Laboratories Inc., Hercules, A, USA). Protein bands of NFATC4 and STIM1 were transfered by electroblotting to a nitrocellulose membrane (Whatman, GE Healthcare, Freiburg, Germany). For PPP3R1 analysis, protein bands were transferred to a polyvinylidene fluoride membrane. Protein bands were detected using specific monoclonal antibodies for STIM1 (anti-STIM1; Cat# 5668 S) and NFATC4 (anti-NFAT3; Cat# 2183 S) from Cell Signaling Technology, Inc. (Danvers, USA) and for PPP3R1 (Cat# MA5-23933) from Thermo Fisher Scientific (Waltham, USA). β-Actin served as loading control and was detected by monoclonal anti-beta-Actin antibody (Cat# A5441) from Sigma Aldrich (Munich, Germany). Secondary antibodies were purchased from Sigma Aldrich.

### Quantification of IL-2 production by ELISA assay

Jurkat E6.1 cells, 2.5 × 10^5^ cells/well were seeded out in six-well plates and transfected with ANC or syn-hsa-miR-34a-5p miScript miRNA Mimics (QIAGEN N.V.) as described for protein extraction. Twenty-four hours after transfection, cells were collected for activation. The transfected cells were transferred to a 48-well plate and seeded out to a count of 3.5 × 10^5^ cells/well in a volume of 350 µl fresh medium, supplemented with MACSiBead™ Particles (Bead:Cell ratio 1:2) from human T Cell Activation/Expansion Kit (Miltenyi Biotec GmbH) and 5 ng/ml of PMA (Sigma Aldrich) was added. Incubation time was additional 24 h before supernatants were collected and total cell count was estimated. IL-2 quantification was performed following the protocol of Human IL-2 DuoSet® ELISA of R&D Systems, Inc. (Minneapolis, Minnesota, USA).

### Statistical evaluation and quantification

Statistical evaluation was performed by Student’s *t*-test, using SigmaPlot 10 software (Systat, Chicago, USA). A normal distribution of data was expected in all experiments. Densitometric analysis of western blot bands was performed, using Image Lab Software Version 5.2.1 (Bio-Rad Laboratories, Inc.). The difference of data sets was considered to be significant at a *p*-value ≤ 0.05. Asterisks in the figures correspond to the statistical significance: *p ≤ 0.05, **p < 0.01, ***p < 0.001.

## Electronic supplementary material

Supplemental informations
