# Supplementary material for: Modulation of intracellular calcium signaling by microRNA-34a-5p
Source: Cell Death Dis. 2018 Sep 27;9(10):1008. doi: 10.1038/s41419-018-1050-7 (PMC6160487; doi:10.1038/s41419-018-1050-7)
Supplement: Supplementary file 1 — Supplemental informations [file 41419_2018_1050_MOESM1_ESM.pdf]

Author contributions

Conceptualization: M.H., C.D., B.A.N., D.A., B.W-R., F.G., H-P.L., A.K., E.M.; Investigation: C.D., D.A., S.R., V.P., M.H.; Formal Analysis: C.D., D.A., M.H.; Writing–Original Draft: C.D., M.H., E.M.; Writing–Review & Editing of text and figures: M.H., C.D., D.A., B.A.N., B.W-R., T.F., J.M., F.G., H-P.L., A.K., E.M.

Figures 1-6, supplementary figures 1-5: C.D. and M.H. plotted the data and prepared the figures.

Supplementary figures and tables:

Supplementary figure 1

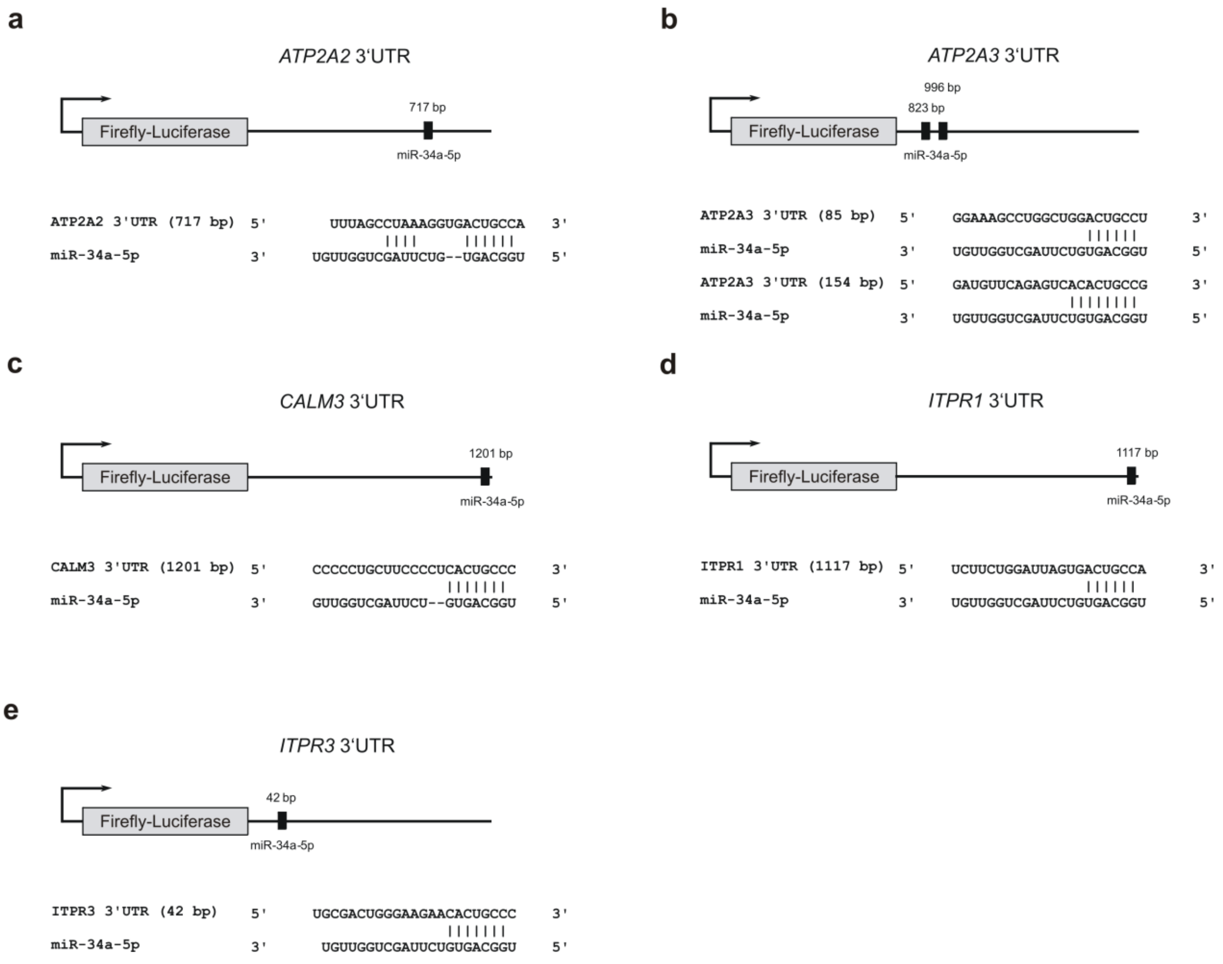

Supplementary figure 1: Schematic representation of reporter gene constructs.

3’UTR sequences of *ATP2A2* (ATPase sarcoplasmic/endoplasmic reticulum Ca^{2+} transporting 2), *ATP2A3* (ATPase sarcoplasmic/endoplasmic reticulum Ca^{2+} transporting 3), *CALM3* (Calmodulin 3), *ITPR1* (inositol 1,4,5-trisphosphate receptor type 1) and *ITPR3* (inositol 1,4,5-trisphosphate receptor type 1) were cloned into pMIR-RNL-TK reporter plasmids. The approximate position of the predicted miR-34a-5p binding sites within the 3’UTR reporter constructs are illustrated and the sequences of the binding sites within the respective 3’UTRs are denoted.

Supplementary figure 2

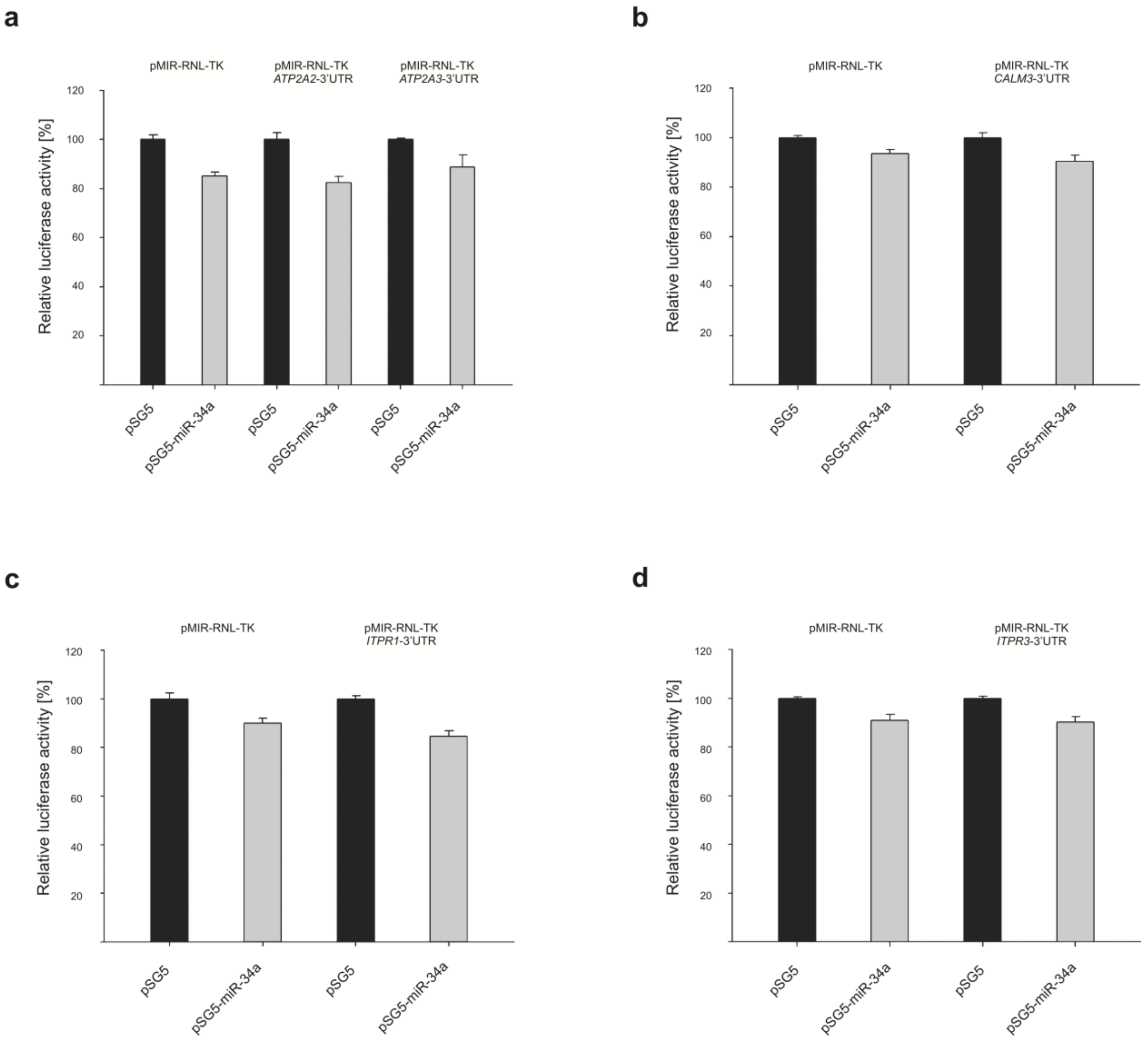

Supplementary figure 2: Results of luciferase assays, showing no impact of miR-34a-5p on predicted target genes related to SOCE or calcineurin/NFAT signaling.

3'UTR sequences of *ATP2A2* (ATPase sarcoplasmic/endoplasmic reticulum Ca2+ transporting 2), *ATP2A3* (ATPase sarcoplasmic/endoplasmic reticulum Ca2+ transporting 3), *CALM3* (Calmodulin 3), *ITPR1* (inositol 1,4,5-trisphosphate receptor type 1) and *ITPR3* (inositol 1,4,5-trisphosphate receptor type 1) were analysed by dual luciferase assays. Relative luciferase activity [%] is shown for empty reporter plasmids (pMIR-RNL-TK) as well as wild type 3'UTR containing constructs. HEK293T cells were co-transfected with control (pSG5) or miR-34a-expression plasmids. Luciferase activities were measured 48 h after transfection. Results are shown as means of 4 independent experiments with corresponding standard errors (SEM). Statistical evaluation was performed using student`s t-test. A normal distribution of the data was assumed. Compared to empty reporter plasmid, results of 3'UTR constructs revealed no statistical significance. A p value of ≤ 0.05 was considered to be significant.

Supplementary figure 3

A

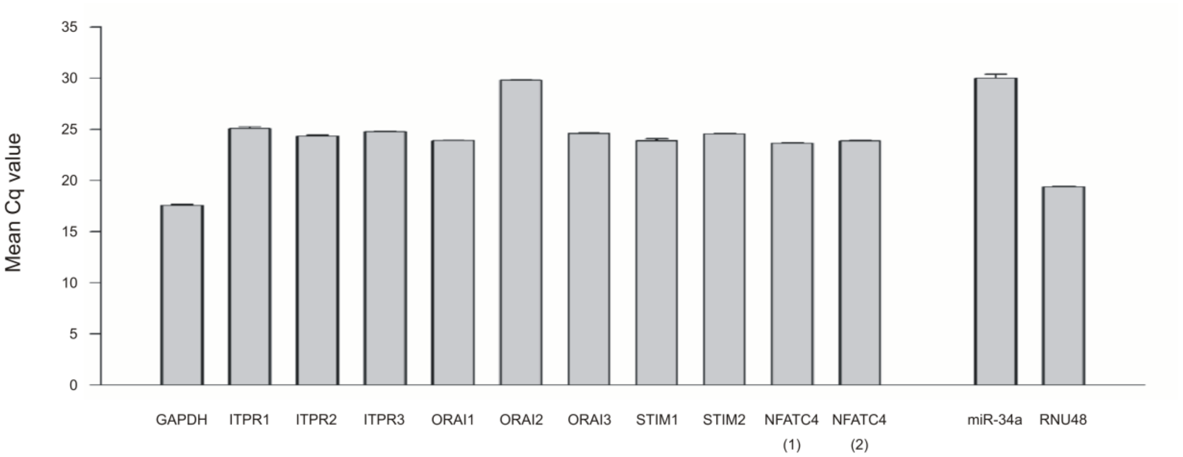

B

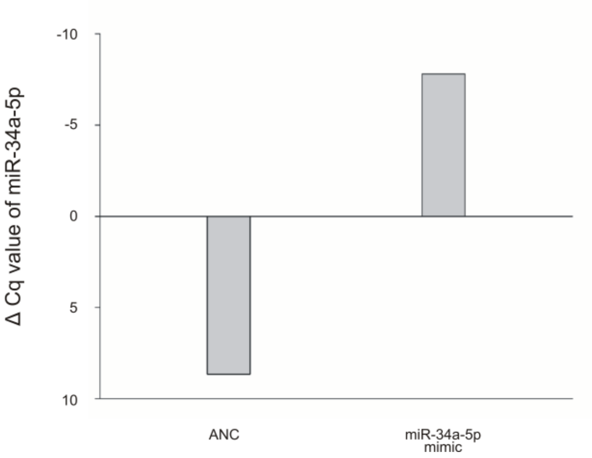

Supplementary figure 3: qRT-PCR analysis of endogenous mRNA levels and microRNA-34a-5p tranfection in Jurkat cells.

A: *ITPR1* (inositol 1,4,5-trisphosphate receptor type 1) and *ITPR3* (inositol 1,4,5-trisphosphate receptor type 1), *ORAI1* (ORAI calcium release-activated calcium modulator 1), *ORAI2* (ORAI calcium release-activated calcium modulator 2), *ORAI3* (ORAI calcium release-activated calcium modulator 3), *STIM1* (Stromal interaction molecule 1), *STIM2* (Stromal interaction molecule 2), *NFATC4* (Nuclear factor of activated T cells 4; detected by two different primer pairs (1), (2)) and *miR-34a* mean quantification cycle (Cq) values are shown as mean with SEM of a triplicate analysis. GAPDH and RNU48 served as endogenous mRNA and microRNA expression control.

B: Jurkat cells were transfected for 48 h either with non-targeting control (ANC) or synthetic miR-34a-5p mimic. RNU48 served as endogenous microRNA expression control. Mean Cq values were calculated based on a triplicate analysis and ΔCq of miR-34a-5p was determined by subtraction of the endogenous control.

Supplementary figure 4

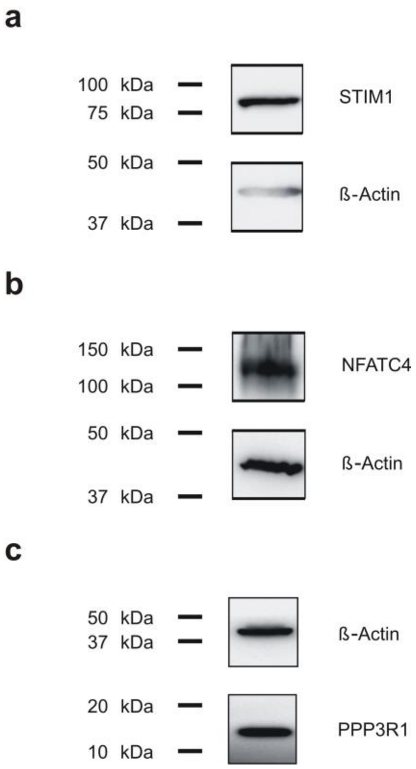

Supplementary figure 4: Western blot analysis of endogenous STIM1 (Stromal interaction molecule 1; A), NFATC4 (Nuclear factor of activated T cells 4; B) and PPP3R1 (Protein phosphatase 3 regulatory subunit B, alpha; C) protein expression in Jurkat cells.

STIM1, NFATC4 and PPP3R1 protein was detected by specific monoclonal antibodies, respectively. β-Actin served as loading control.

Supplementary figure 5

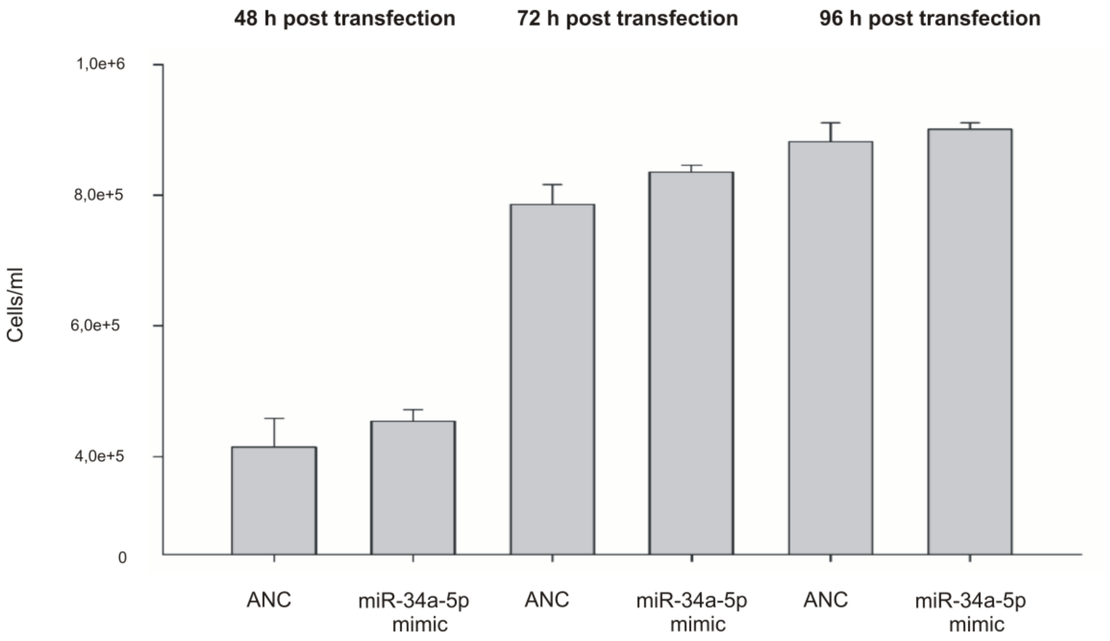

Supplementary figure 5: Cell counts of Jurkat cells after microRNA-34a-5p transfection.

Jurkat cells were transfected either with non-targeting control (ANC) or miR-34a-5p mimic for different periods of time. Corresponding cell counts were determined and are shown as means of three independent transfections with corresponding standard errors (SEM). Statistical evaluation was performed using student`s t-test. A normal distribution of the data was assumed. No significant differences were detected.

Supplementary table 1: List of oligonucleotide primer pairs including added enzyme restriction sites.

The amplified 3’UTR sequences of predicted miR-34a-5p target genes were cloned into pMIR-RNL-TK for luciferase gene reporter analysis.

Predicted target gene	Strand	Cloning primer sequence (5'→3')	Restriction site
ATP2A2	forward	GACTAGTGTATGCCTGTACTCGCTTGTG	*SpeI*
	reverse	CGAGCTCGTCTGCACAGCTCCTTCAG	*SacI*
ATP2A3	forward	GACTAGTGTCAGAGAGACGGACACAAG	*SpeI*
	reverse	GACTAGTCTCTACCAAACAGCAGGTCAG	*SpeI*
CALM3	forward	GACTAGTCCAAGCTGCATGATTGCTC	*SpeI*

	reverse	CGAGCTCGGGTGGATGAGAAGAGATTC	*SacI*
CAMLG	forward	GACTAGTGCCTGTAGAACTGAGAAGGAG	*SpeI*
	reverse	CGAGCTCGGAAATGTAACACCAAGTGAC	*SacI*
ITPR1	forward	GACTAGTCCCGAGTGTCCTAAAGGGAGTG	*SpeI*
	reverse	CGAGCTCCCAGTAAAAGCAGAAGAGACAGGAG	*SacI*
ITPR2	forward	GACTAGTCAGGATGTGATTGCCTTCTC	*SpeI*
	reverse	CGAGCTCGGATCATGAATGTCAGGGTC	*SacI*
ITPR3	forward	GACTAGTCAACAGGGGATGCTCATCAC	*SpeI*
	reverse	CGAGCTCGCCAAAATGTAGTGCTTGGAG	*SacI*
NFATC4	forward	GACTAGTCAGCACATTGAACGCTGCACT	*SpeI*
	reverse	CGAGCTCGCTGTTTAATAAATATCTGACAATGCATG	*SacI*
ORAI3	forward	GACTAGTCGCTACAAGCAGGAACTAGAGG	*SpeI*
	reverse	CGAGCTCCAACCCTTGCTAAGGACTCAG	*SacI*
PPP3R1	forward	GACTAGTCTGCTTGAGGAGAAAGTCTAG	*SpeI*
	reverse	CGAGCTCGGCTAGTTCCCCCTTGAAG	*SacI*
RCAN1	forward	GACTAGTGGAGAGAGCTTCCTGTTTC	*SpeI*
	reverse	CGAGCTCCCTATTTACCAGACATACAC	*SacI*
STIM1	forward	GACTAGTGACGAGTAGCTTCTGACATG	*SpeI*
	reverse	CGAGCTCCTCCTACATGCTTTATTGGAC	*SacI*

Supplementary table 2: List of oligonucleotide primer pairs for (primary) overlap extension PCR.

MiR-34a-5p binding sites within the 3'UTR sequences of miR-34a-5p target genes were replaced with enzyme restriction sites by overlap extension PCR and cloned into pMIR-RNL-TK. Wildtype 3'UTR-pMIR-RNL-TK reporter constructs were used for template. In primary overlap extension PCR two overlapping mutated sequences were amplified that were used for template in secondary PCR (in combination with cloning primer pairs) to generate the complete mutated 3'UTR insert.

* Oligonucelotide was used for whole amplicon amplification, no secondary PCR was required.

Target gene	Strand	Mutagenesis primer sequence (5'→3')	Restriction site	Combined primer sequence (5'→3') for primary PCR
ORAI3	forward	CACCGCTCACTGCAAGT	*NruI*	CCAAGCTAGCGGCCGCATACAA (pMIR)

		CGCGAGTCCCTCCGGGGTCTG		
	reverse	CAGACCCCGGAGGGACTCGCGACTTGCAGTGAGCGGTG	*NruI*	GAAGTACCGAAAGGTCTTACCG (pMIR)
	forward	GGATGGTGCCTGACCAGCGCTGCTAGCCCCACGCTATG	*AfeI*	CCAAGCTAGCGGCCGCATACAA (pMIR)
	reverse	CATAGCGTGGGGCTAGCAGCGCTGGTCAGGCACCATCC	*AfeI*	GAAGTACCGAAAGGTCTTACCG (pMIR)
CAMLG	forward	CTTTTAACTAACTCAGTATCGCGAATATTTTGTTGGGTTG	*NruI*	CCAAGCTAGCGGCCGCATACAA (pMIR)
	reverse	CAACCCAACAAAATATTCGCGATACTGAGTTAGTTAAAAG	*NruI*	GACTAGTGCCTGTAGAACTGAGAAGGAG
ITPR2	forward	CCCACAGGAACAAAATCGCGAGTCCAGAGTCTTTAAATTC	*NruI*	CGAGCTCGGATCATGAATGTCAGGGTC
	reverse	GAATTTAAAGACTCTGGACTCGCGATTTTGTTCCTGTGGG	*NruI*	GACTAGTCAGGATGTGATTGCCTTCTC
NFATC4	forward	GCCCCCTGCCAGAGTTCGCGAGCCATAATCACCATGTC	*NruI*	CGAGCTCGCTGTTTAATAAATATCTGACAATGCATG
	reverse	GACATGGTGATTATGGCTCGCGAACTCTGGCAGGGGGC	*NruI*	GACTAGTCAGCACATTGAACGCTGCACT
	reverse*	CGAGCTCGCTGTTTAATAAATATCTGACAATGCA	*SacI* *(cloning),*	GACTAGTCAGCACATTGAACGCTGCACT

		TGAATCAGCGCTTCCTGGCAGAAAACAG	*AfeI*	
PPP3R1	forward*	GACTAGTCTGCTTGAGGAGAAAGTCTAGTTCATTGCTTCGCGAAAGAGCTAGTTCTTG	*SpeI* (cloning), *NruI*	CGAGCTCGGCTAGTTCCCCCTTGAAG
	reverse*	CGAGCTCGGCTAGTTCCCCCTTGAAGCAGAGCACATAGCGCTTGACACTGGAACAG	*SacI* (cloning), *AfeI*	GACTAGTCTGCTTGAGGAGAAAGTCTAG
RCAN1	forward	CAGAGAATAGCAGTTTTCGCGAGGACTTTTAGTGGGTGAG	*NruI*	CGAGCTCCCTATTTACCAGACATACAC
	reverse	CTCACCCACTAAAAGTCCTCGCGAAAACTGCTATTCTCTG	*NruI*	GACTAGTGGAGAGAGCTTCCTGTTTC
STIM1	forward	GGTGCTAGGCTGCAGAATATTTTGTACTCCCTGGAC	*SspI*	CCAAGCTAGCGGCCGCATACAA (pMIR)
	reverse	GTCCAGGGAGTACAAAATATTCTGCAGCCTAGCACC	*SspI*	GACTAGTGACGAGTAGCTTCTGACATG
